# Metagenetic Analysis for Microbial Characterization of *Focaccia* Doughs Obtained by Using Two Different Starters: Traditional Baker’s Yeast and a Selected *Leuconostoc citreum* Strain

**DOI:** 10.3390/foods10061189

**Published:** 2021-05-25

**Authors:** Massimo Ferrara, Angelo Sisto, Giuseppina Mulè, Paola Lavermicocca, Palmira De Bellis

**Affiliations:** Institute of Sciences of Food Production (ISPA), National Research Council (CNR), Via G. Amendola 122/O, 70126 Bari, Italy; massimo.ferrara@ispa.cnr.it (M.F.); angelo.sisto@ispa.cnr.it (A.S.); giuseppina.mule@ispa.cnr.it (G.M.); paola.lavermicocca@ispa.cnr.it (P.L.)

**Keywords:** yeast-free dough, baker’s yeast, liquid sourdough, metagenetic analysis, lactic acid bacteria, microbiota

## Abstract

Lactic acid bacteria (LAB) decisively influence the technological, nutritional, organoleptic and preservation properties of bakery products. Therefore, their use has long been considered an excellent strategy to improve the characteristics of those goods. The aim of this study was the evaluation of microbial diversity in different doughs used for the production of a typical Apulian flatbread, named *focaccia.* Leavening of the analyzed doughs was obtained with baker’s yeast or by applying an innovative “yeast-free” protocol based on a liquid sourdough obtained by using *Leuconostoc citreum* strain C2.27 as a starter. The microbial populations of the doughs were studied by both a culture-dependent approach and metagenetic analyses. The flours used for dough preparation were also subjected to the same analyses. The metagenetic analyses were performed by sequencing the V5–V6 hypervariable regions of the 16S rRNA gene and the V9 hypervariable region of the 18S rRNA gene. The results indicate that these hypervariable regions were suitable for studying the microbiota of doughs, highlighting a significant difference between the microbial community of *focaccia* dough with baker’s yeast and that of the dough inoculated with the bacterial starter. In particular, the dough made with baker’s yeast contained a microbiota with a high abundance of *Proteobacteria* (82% of the bacterial population), known to be negatively correlated with the biochemical properties of the doughs, while the *Proteobacteria* in dough produced with the *L. citreum* starter were about 43.5% lower than those in flour and dough prepared using baker’s yeast. Moreover, the results show that the *L. citreum* C2.27 starter was able to dominate the microbial environment and also reveal the absence of the genus *Saccharomyces* in the dough used for the production of the “yeast-free” *focaccia*. This result is particularly important because it highlights the suitability of the starter strain for obtaining an innovative “yeast-free” product.

## 1. Introduction

Traditionally, dough leavening of bakery products has been obtained using sourdough, but with the development of industrial baking at the beginning of the 20th century, baker’s yeast (*Saccharomyces cerevisiae*) almost completely replaced sourdough as a more rapid leavening agent, which also occurred in several traditional productions [1]. In fact, baker’s yeast is a ready-to-use starter able to produce high amounts of CO_2_ and flavor compounds [2,3]. The typical Apulian *focaccia* flatbread has recently been included in the Italian list of unprotected typical/local products (PAT, Prodotti Agroalimentari Tradizionali) [4]. Its production is characterized by a fermentation process using baker’s yeast on a blend of soft and durum wheat flours and water, extra-virgin olive oil and salt [5]. The nutritional values of a commercial Apulian *focaccia* (100 g) are approximately the following: 305 kcal, carbohydrates 44.28 g of which sugars 2.39 g, fats 11 g of which saturates 1.36 g, proteins 6.9 g, salt 0,74 g [6].

Although the use of baker’s yeast as a starter shortens the leavening time, it does not provide products with high-quality standards [7,8]. Therefore, the use of sourdough is once again increasing, especially in retail or artisanal bakeries, due to its characteristics [9,10]. In fact, sourdough fermentation may improve the functional/nutritional features of leavened baked goods by lowering the glycemic index, increasing mineral bioavailability, decreasing the gluten content, masking the decreased salt content and/or enriching bakery products with functional antihypertensive compounds [11,12], in addition to determining a better flavor, an extended shelf life and a reduction in additives [13]. The abundant studies in the literature show the features of sourdough fermentation and the need to develop new products based on the use of selected or novel sourdoughs and on targeted processes in relation to the desired characteristics of food products [12,14,15]. In fact, sourdough is a mixture of flour and water, fermented with lactic acid bacteria (LAB) and yeast, and the traditional sourdough fermentation is generally a long process involving interactions among many factors such as microbiota and related metabolic activities, fermentation parameters and raw materials [7,16]. Sourdough standardization is difficult due to its complexity, and this constitutes a problem for the bakery industry, which requires product stability and reproducibility, and short leavening times [15]. To overcome these limitations, liquid sourdough (type II) fermentation with single or mixed starter cultures has recently been introduced in bakeries [13,15,17,18]. Liquid sourdough offers several advantages compared to traditional sourdough, such as great flexibility of use, easier fermentation control, easier management and reproducibility [7]. Moreover, tailored biotechnological protocols can be used to modify the nutritional and/or functional features of the products. In fact, the growing consumer interest in foods with improved nutritional and functional quality has prompted food producers to adapt protocols for upgrading product attributes, including those of traditional products [19]. Product labels often include nutritional claims, such as “low fat”, “high fiber”, “light”, “sugar-free”, “source of protein” and “low salt”. Similarly, there is an increasing interest of the market in “yeast-free” leavened foods, due to the recent concern about the role of *S. cerevisiae* (particularly its anti-antibodies) in several pathologies, including Crohn’s disease, gastro-esophageal reflux, acne inversa and gut fermentation syndrome [20,21,22,23]. In this context, processes to develop “yeast-free” bakery products have been formulated and applied at the industry level [24,25,26]. In particular, we developed a protocol based on the use of a liquid sourdough started with a single bacterial strain for “yeast-free” bread and *focaccia* production, obtaining products with improved functional/nutritional characteristics compared to those made with baker’s yeast [24,27]. The strain *L. citreum* C2.27 was selected for its technological characteristics, particularly its leavening capacity, thus allowing the production of doughs without using baker’s yeast.

The aim of this study was to characterize the bacterial and fungal microbiota of doughs used for *focaccia* production made with liquid sourdough inoculated with *L. citreum* C2.27 or with baker’s yeast. The microbial community was investigated by using culture-dependent and culture-independent approaches. Several studies have investigated the bacterial microbiota associated with traditional sourdough [28,29], but only a few metagenetic studies have been concerned with the characterization of the fungal microbiota of sourdoughs [30,31,32]. The absence of metagenetic studies on fungal communities is probably due to non-standardized metagenetic analysis of yeast, including selection of the optimal region to amplify [33,34], or to a lower general interest in the yeast composition [35]. To our knowledge, this is the first study that explores, also using an in-depth metagenetic approach, the bacterial and fungal microbiota of doughs leavened with baker’s yeast or with a type II sourdough (started with a selected bacterial strain) used to obtain an innovative “yeast-free” product.

## 2. Materials and Methods

### 2.1. Bacterial Starter Strain and Culture Conditions

The strain *L. citreum* C2.27 (ITEM 17404) belongs to the ITEM Culture Collection of the Institute of Sciences of Food Production of the National Research Council (ISPA-CNR) and was isolated from Italian durum wheat semolina [36]. The cell culture was routinely propagated (2% *v*/*v*) in de Man Rogosa Sharpe (MRS) broth (Oxoid Ltd., Basingstoke, UK) and incubated at 30 °C for 24 h.

### 2.2. Liquid Sourdough and Dough Fermentation

Two different doughs were used for the production of *focaccia*: (a) dough using the liquid sourdough started with lactic acid bacteria; (b) dough using baker’s yeast as described below. *L. citreum* C2.27, which belongs to a species included in the list of biological agents with Qualified Presumption of Safety (QPS) [37], was used as starter to produce the liquid sourdough as previously described [24].

**Figure 1:**
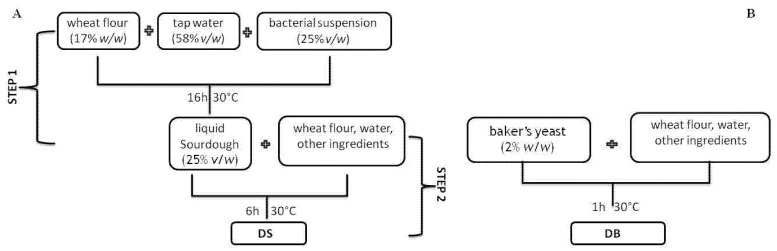
Schematic representation of the production of two different doughs: (**A**) dough with the liquid sourdough started with *L. citreum* C2.27 (DS), and (**B**) dough with baker’s yeast (DB).

Liquid sourdough (S) (500 g) was prepared by mixing wheat flour (17% *w*/*w*), sterile tap water (58% *v*/*w*) and bacterial suspension (25% *v*/*w*). The cell density in the sourdough was ca. 8 log cfu/g and dough yield (DY) was 600. The mixture was incubated at 30 °C for 16 h (Figure 1).

*Focaccia* dough (DY ca. 172) with liquid sourdough (DS) was prepared by mixing durum wheat semolina (27% *w*/*w*, Divella, Rutigliano, ITA), soft wheat flour type “00” (27% *w*/*w*; Casillo, Corato, ITA), tap water (18.36% *v*/*w*), extra-virgin olive oil (2.1% *v*/*w*; Agridè, Bitonto, ITA), malt barley flour (0.54% *w*/*w*; Antico Molino Rosso, Buttapietra, ITA) and S (25% *v*/*w*). DS was compared with *focaccia* dough (DB) prepared using baker’s yeast (2% *w*/*w*, corresponding to a final yeast density of ca. 8 log cfu/g) and without liquid sourdough. Three independent tests were carried out. Dough portions of 180 g were placed in round non-stick pans and fermented at 30 °C for 6 h for DS or 1 h for DB. After fermentation, the microbial communities were analyzed, together with the pH drop (ΔpH, pH units) and total titratable acidity (TTA, ml di NaOH 0.1 N/10 g) for S, DS and DB [30].

### 2.3. Microbiological Analyses of Doughs

After fermentation, liquid sourdough (S) was immediately subjected to decimal dilutions and plating, while 20 g aliquots of *focaccia* doughs (DS and DB) or of a mixture (1:1 ratio) of durum wheat semolina and soft wheat flour type “00” (F) were each homogenized with 180 mL of sterile NaCl solution (0.85%, *w*/*v*) in a Stomacher (Seward, London, UK) for 2 min. After serial dilutions, the microbial suspensions were plated on an mMRS agar (Oxoid, UK) [38] supplemented with 100 mg/l of cycloheximide (EMD Millipore Corp., Billerica, MA, USA) for the determination of lactic acid bacteria (LAB), and on a Sabouraud Dextrose Agar (SDA, Oxoid, UK) supplemented with 200 mg/l chloramphenicol (Sigma, Milan, Italy) for the enumeration of yeasts and molds. Moreover, an aliquot of each microbial suspension was heat treated for 20 min at 90 °C, plated on a Plate Count Agar (PCA, Difco, Franklin Lakes, NJ, USA) and incubated for 24 h at 30 °C for spore-forming bacteria counts. A total of 20% of the colonies from the countable mMRS agar and SDA plates (incubated at 30 °C for 48 h and at 25 °C for 72 h, respectively) were randomly taken, purified and stored at −80 °C.

### 2.4. Characterization and Identification of LAB and Yeasts

Bacterial DNA was extracted from overnight cultures grown in mMRS broth (Oxoid, UK) at 30 °C, using a Clonsaver Card Kit (Whatman, Maidstone, UK) and analyzed by Repetitive Extragenic Palindromic-PCR (REP-PCR) [39]. The identification of *L. citreum* C2.27 was based on the comparison of its strain-specific REP-PCR profile with that of each LAB isolate from liquid sourdough and doughs. Bacterial isolates showing an REP-PCR profile different from that of the starter strain were identified by the sequencing of the almost complete 16S rRNA gene as previously described [24,39], using an ABI Prism 3730 × l DNA Analyzer (Thermo Fisher Scientific, Waltham, MA, USA). The species *Lactiplantibacillus* *paraplantarum* (basonym, *Lactobacillus paraplantarum*) [40] and *Lacticaseibacillus* *paracasei* (basonym *Lactobacillus paracasei*) were also identified by multiplex-PCR methods as described by Torriani et al. [41] and Ventura et al. [42], respectively.

The DNA of yeast isolates was extracted from 1.5 mL cultures grown in YEPG (Yeast Extract 1% *w*/*v*, Peptone 1% *w*/*v* and Dextrose 2% *w*/*v*) at 25 °C for 24 h, using the Wizard Genomic DNA Purification kit (Promega Corporation, Madison, WI, USA), and amplified by the oligonucleotide (GTG)_5_ [43]. The isolates were identified by amplification and sequencing of the D1/D2 domain of the 26S rDNA using the primers NL1 and NL4 [44]. Bacterial and yeast strains were assigned to the species on the basis of the highest scores of alignment and percentage of identity (>99%) between their 16S rRNA/26S rRNA gene sequences and those of type strains in the NCBI Nucleotide database [24].

### 2.5. Culture-Independent Community Identifications

Ninety milliliters of saline solution was added to 10 g of flour mixture (F) or doughs DS and DB and homogenized for 3 min. Homogenates were centrifuged (1000× *g* for 5 min at 4 °C) and the supernatants were recovered and centrifuged (5000× *g* for 15 min at 4 °C). Each pellet was suspended in 0.5 mL of saline solution and the suspension was subjected to DNA extraction. Total genomic DNA was extracted using the FastDNA Pro Soil-Direct kit (MPBiomedicals, Santa Ana, CA, USA) coupled to the Retsch MM301 instrument (Retsch Gmbh, Germany), according to the manufacturer’s instructions. Quality and quantity of DNA extracts were estimated using NanoDrop ND1000 (NanoDrop Technologies, Inc., Wilmington, NC, USA) and by 1% (*w*/*v*) agarose gel electrophoresis in TAE buffer. DNA extraction was carried out in triplicate on each sample.

### 2.6. Library Preparation and Sequencing

The total DNA extracted from the flour mixture and dough samples was used as template for 16S and 18S metagenetic analyses. All DNA samples were equalized at a final concentration of 10 ng/μL for NGS library preparation. Library preparation was performed using a bidirectional fusion primer set to specifically amplify the target regions. This was accomplished by combining primers targeting the regions of interest to the Ion Torrent sequencing adapters Ion A and truncated P1 (trP1). Adapter A included unique Ion Xpress Barcode sequences for sample multiplexing. Bidirectional sequencing was achieved by swapping adapter sequences A and trP1. Therefore, the V5–V6 hypervariable region of the16S rRNA gene was amplified with primers 785F (GGATTAGATACCCTGGTA) and 1100R (GGGTTGCGCTCGTTG). For 18S libraries, the V9 hypervariable region was amplified with primers 1380F (CCCTGCCHTTTGTACACAC) and 1510R (CCTTCYGCAGGTTCACCTAC). The PCR reactions were carried out in triplicates using Platinum SuperFi DNA Polymerase (Invitrogen, USA) with the following conditions: 98 °C for 30 min, 25 cycles of 98 °C for 10 s, 58 °C for 20 s and 72 °C for 30 s and a final extension of 72 °C for 5 min. Amplified libraries were verified on 2% agarose gel, and PCR products were purified using Agencourt AMPure XP magnetic beads (Agencourt Bioscience, Beverly, MA, USA). Purified libraries were quantified with Qubit dsDNA HS Assay Kit (Invitrogen, Carlsbad, CA, USA) and pooled at a final concentration of 100 pM. Libraries were sequenced on an Ion S5 Sequencing System (Thermofisher, Waltham, MA, USA). Following the sequencing run, a FASTQ file was generated for each sample and demultiplexed by using the built-in software of Torrent Suite.

### 2.7. Bioinformatics

Quality-filtered reads were aligned using MALT (v0.4.1) [45], in BlastN mode, against the SILVA ribosomal RNA sequence database (SSURef_NR99, release 128), with default settings and alignment type set to “SemiGlobal”. For taxonomic binning, MEGAN software (MEGAN 6 v6.18.1) [46] was used. For 16S libraries, the LCA algorithm was set with the following settings: minScore = 500, maxExpected = 1.0, minIdentity= 0.01, topPercent = 10 and minSupport = 10. For 18S libraries, the LCA algorithm was set with the following settings: minScore = 250, maxExpected = 1.0, minIdentity= 0.01, topPercent = 10 and minSupport = 10. Sequences were assigned at the taxonomic level with at least 97% identity for genus and 99% for species in the reference database.

### 2.8. Statistics

Analysis of variance (ANOVA) combined with the Tukey–Kramer method as a post hoc test was applied for microbiological analysis. Significant differences (*p* < 0.05) among *focaccia* dough samples are marked with different letters. Rank abundance and alpha and beta diversity indexes were estimated using Microbiome Analyst [47]. Moreover, principal coordinate analysis (PCoA) was calculated using the Bray–Curtis index. Hypothesis testing was conducted by the analysis of molecular variance (AMOVA) test (*p* < 0.05) [48]. Statistical comparison among taxonomic categories was performed using STAMP software [49]. Differences between groups were analyzed using Welch’s t-test (*p* < 0.05) [50], and the Benjamini–Hochberg false discovery rate (FDR) method (q-value < 0.05) was used [51].

### 2.9. Data Availability

The sequence data are available at NCBI SRA under BioProject ID: PRJNA668040.

## 3. Results

### 3.1. Culture-Dependent Microbiological Analyses

Durum wheat semolina and soft wheat flour type “00” in a 1:1 ratio (F) and, at the end of fermentation, S, DS and DB doughs were subjected to microbiological analyses to assess the cell load of LAB, yeasts, molds and spore-forming bacteria (Table 1). The use of sourdough influenced the pH values of DS, which, after fermentation, showed pH 4.2 and ΔpH 1.3, while DB presented a pH value (5.38) that was significantly higher (*p* < 0.05). Moreover, S showed pH and TTA values lower than DS. This apparently contrasting result is due to the higher value of DY in S than in DS. In fact, it is known that a low DY value, as in DS, increases the buffering capacity of the flour, lowering the rate of acidification, even if organic acids are present at higher levels [52].

S and DS showed cell densities of LAB (9 log cfu/g) that were significantly higher than DB (4 log cfu/g). LAB isolates from liquid sourdough and doughs (20% of the colonies from countable plates) were characterized by REP-PCR. The analysis of their electrophoretic profiles revealed the presence of only the starter strain in S and DS, while four LAB strains were detected in DB, belonging to *Leuconostoc mesenteroides*, *Furfurilactobacillus* *rossiae* (basonym *Lactobacillus rossiae*), *Lp. paraplantarum* and *Lc. paracasei*. These species are typically contained in sourdough [17] and are among the species found in doughs made with different flours from the same geographical area [24]. In addition to the absence of LAB strains isolated from DB, the only strain isolated directly from the flours, belonging to *Loigolactobacillus* *coryniformis* (basonym *Lactobacillus coryniformis*), was also absent in DS. It is probable that this strain was no longer found in the doughs because it was dominated during fermentation by the starter strain or by the other strains, the latter evidently present in the flours in quantities lower than the detection limit.

The presence of spore-forming bacteria in the doughs was also evaluated. Bacterial counts were very low (<1 log cfu/g) and therefore below the quantities which could cause product alteration (ropy bread) (≥2 log cfu/g) or constitute a risk for consumer health (>5 log cfu/g) [53].

Yeasts were absent in S and DS, while molds were present in very low quantities. On the contrary, DB contained a high density of yeasts (8.25 log cfu/g), while molds were not detected. The REP-PCR analysis of the yeasts showed the presence of only one strain belonging to *S. cerevisiae*. Finally, only one yeast strain belonging to *Cryptococcus victoriae* was isolated from the flours but was not subsequently found in the doughs, probably due to the characteristics of the ecosystem and the presence of the starters. Overall, the results show the capacity of the *L. citreum* C2.27 strain to dominate the microbiota in DS, and, at the same time, the ability of *S. cerevisiae* to dominate the mycobiota in DB.

### 3.2. Bacterial and Fungal Microbiota in Flour and Doughs

After quality filtering, a total of 3,201,028 and 7,194,310 reads were generated and 3,055,906 (95.5%) and 6,648,063 (92.4%) were assigned to a taxon for 16S and 18S, respectively.

**Figure 2:**
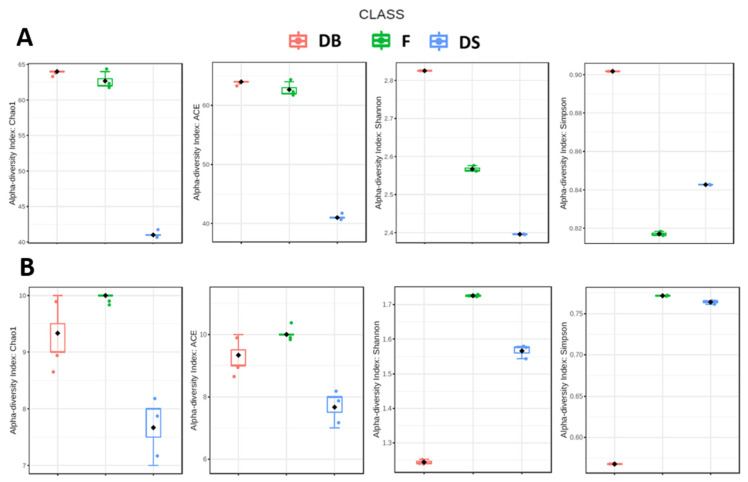
Alpha diversity indexes (Chao1, ACE, Shannon, Simpson) of bacterial (**A**) and fungal (**B**) microbiota in dough with baker’s yeast (DB), dough with sourdough (DS) and flour (F) samples.

The abundance-based coverage estimators (ACE) and the alpha diversity index Chao1 indicated that the richness of the samples was DB > F > DS (Figure 2A). The inoculum of *L. citreum* reduced the abundance of OTUs, if compared with DB and F samples. The loss of OTUs also reduced both richness (Chao1 and ACE) and diversity (Shannon and Simpson) indexes in DS samples, indicating that the use of *L. citreum* as a bacterial starter had a strong effect on microbial community diversity. Considering the same population indexes, even the mycobiota diversity was strongly influenced by the presence of *S. cerevisiae* in the DB sample (Figure 2B).

**Figure 3:**
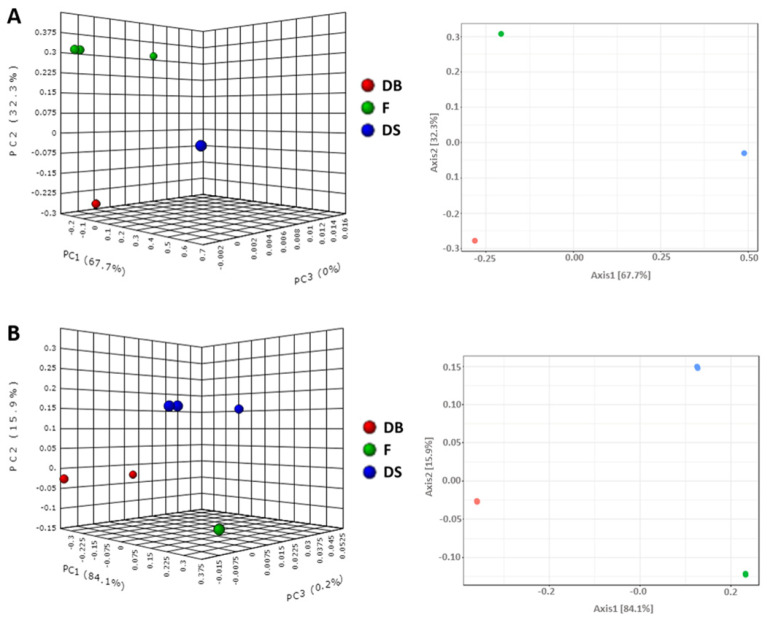
Principal coordinate analysis (PCoA) as 2D (right) and 3D (left) plots of bacterial (**A**) and fungal (**B**) communities of dough with baker’s yeast (DB), dough with sourdough (DS) and flour (F) samples. PCoA was calculated using the Bray–Curtis index to compute dissimilarities among different samples. Hypothesis testing was conducted by analysis of molecular variance (AMOVA) test (*p* < 0.05).

The PCoA analysis showed that the microbial communities in DB, DS and F samples were distinct from each other. The sample triplicates resemble each other very closely (Figure 3). The results were also validated with analysis of molecular variance (AMOVA). This confirmed that the inoculum of *L. citreum* or *S. cerevisiae* significantly affected microbial consortia in DS and DB samples, respectively.

**Figure 4:**
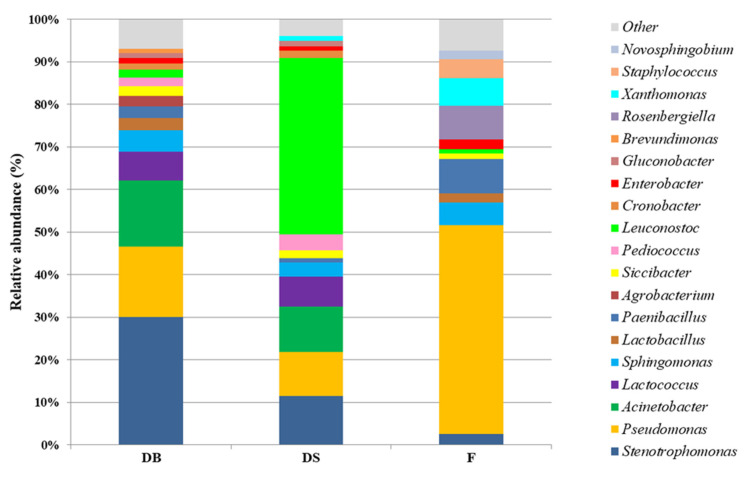
Relative abundance and taxonomic assignments of microbial flora at genus level of dough with baker’s yeast (DB), dough with sourdough (DS) and flour (F) samples. Minor genera with <0.1% relative abundance are represented as “Other”.

Considering the absolute number of assigned reads, the phyla of *Proteobacteria* (1,508,548 assigned reads) and *Firmicutes* (1,322,874 assigned reads) were the most representative in all the samples. The DB microbial consortium was composed mainly of ten different genera belonging to the *Proteobacteria* phylum. *Stenotrophomonas* (relative abundance of 30.0%) was predominant, followed by *Pseudomonas* (16.6%), *Acinetobacter* (15.5%), *Sphingomonas* (5.0%), *Agrobacterium* (2.5%), *Siccibacter* (2.3%), *Cronobacter* (1.4%), *Enterobacter* (1.3%), *Gluconobacter* (1.2%) and *Brevundimonas* (1.0%). Within the *Firmicutes* phylum, the relative abundance was mainly represented by *Lactococcus* (6.8%), *Lactobacillus* (2.9%), *Paenibacillus* (2.7%), *Pediococcus* (2.0%) and *Leuconostoc* (1.9%). From the DS sample, the genera belonging to the *Proteobacteria* phylum were *Stenotrophomonas* (11.5%), *Acinetobacter* (10.7%), *Pseudomonas* (10.3%), *Sphingomonas* (3.2%), *Siccibacter* (2.0%), *Cronobacter* (1.7%), *Gluconobacter* (1.2%), *Enterobacter* (1.1%) and *Xanthomonas* (1.1%). Within *Firmicutes*, the genus *Leuconostoc* (41.4%) was dominant, followed by *Lactococcus* (7.1%), *Pediococcus* (3.7%), *Paenibacillus* (1.0%) and *Lactobacillus* (<1%). From the F sample, the genera belonging to the *Proteobacteria* phylum were *Pseudomonas* (49.1%), *Rosenbergiella* (7.9%), *Xanthomonas* (6.4%), *Sphingomonas* (5.4%), *Stenotrophomonas* (2.5%), *Enterobacter* (2.3%), *Novosphingobium* (2.0%) and *Siccibacter* (1.3%). Within *Firmicutes, Paenibacillus* (8.0%), *Staphylococcus* (4.4%), *Lactobacillus* (2.1%) and *Leuconostoc* (1.1%) were detected (Figure 4).

**Figure 5:**
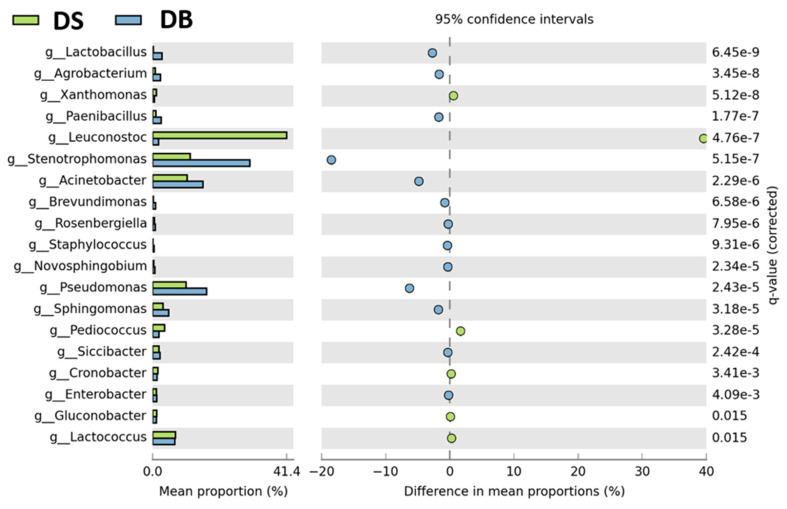
Genera with a significantly different abundance between dough with sourdough (DS) and dough with baker’s yeast (DB) samples. The statistical cutoffs of *p* < 0.05 (Welch test) and *q-value* < 0.05 (FDR) were set as the significance threshold.

**Figure 6:**
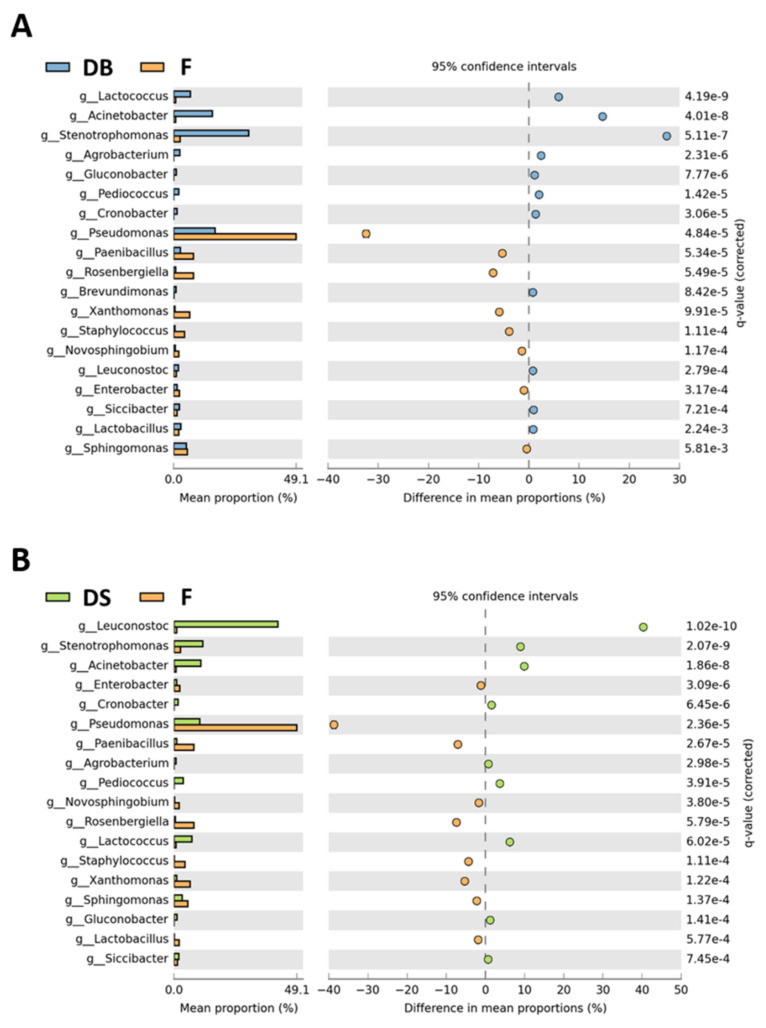
Genera with a significantly different abundance between dough with baker’s yeast (DB) and flour (F) samples (**A**) and dough with sourdough (DS) and flour (F) samples (**B**). The statistical cutoffs of *p* < 0.05 (Welch test) and *q-value* < 0.05 (FDR) were set as the significance threshold.

The relative abundances of identified genera were further compared between sample groups, as shown in Figure 5 and Figure 6.

In addition to the dominance of the genus *Leuconostoc* (relative abundance 39.6% higher than in DB), DS showed a reduction in abundance of 18.5% for *Stenotrophomonas*, 6.3% for *Pseudomonas*, 4.8% for *Acinetobacter* and 1.7% for *Paenibacillus*, compared to DB. Furthermore, within LAB, *Lactobacillus* was significantly lower and *Pediococcus* was significantly higher in DS than in DB (Figure 5). In terms of relative abundance, flour (F) had significantly higher percentages of genera *Pseudomonas* (up to 39%), *Paenibacillus*, *Enterobacter* and *Xanthomonas* than both doughs (Figure 6), while *Sphingomonas* and *Lactobacillus* were higher in F than in DS. *Novosphingobium, Rosenbergiella* and *Staphylococcus* genera were present only in F.

**Figure 7:**
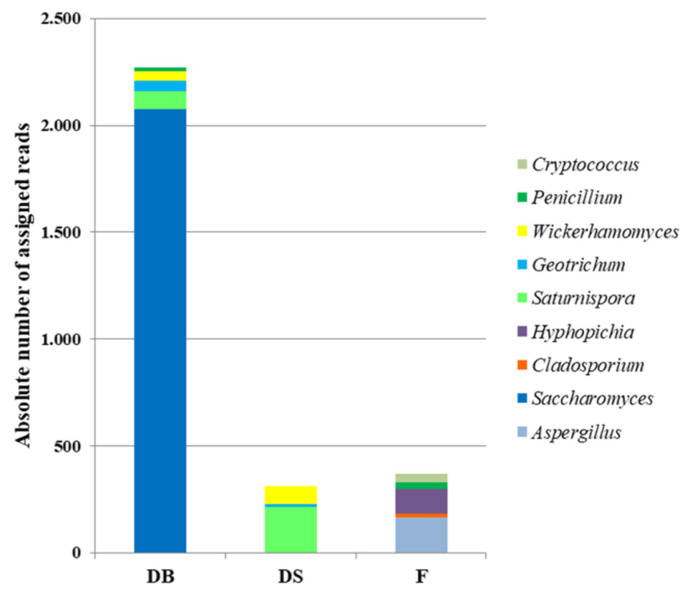
Absolute number of assigned reads to genera belonging to Ascomycota and Basidiomycota phyla for dough with baker’s yeast (DB), dough with sourdough (DS) and flour (F) samples.

**Figure 8:**
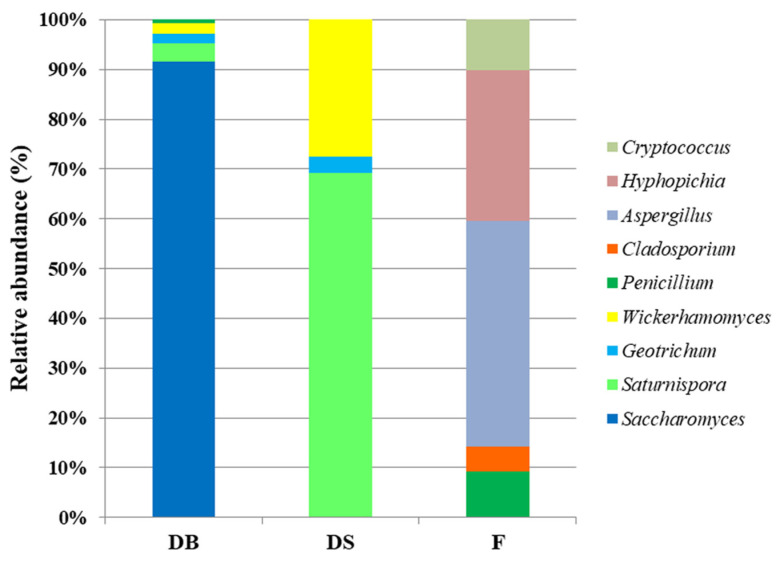
Relative abundance of genera belonging to *Ascomycota* and *Basidiomycota* phyla for dough with baker’s yeast (DB), dough with sourdough (DS) and flour (F) samples. Data are represented as a normalized percentage of the assigned genera.

The metagenetic analysis of mycobiota revealed that the *Saccharomyces* genus dominated the mycobiota in DB and was not detected in other samples (Figure 7 and Figure 8). Considering the absolute number of reads assigned to a taxon, in DB, the overall number of reads assigned to the *Ascomycota* phylum was 6805, while it was 933 in DS and 992 in F. In detail, in the DB samples, 91.5% of reads were assigned to the *Saccharomyces* genus, followed by *Saturnispora* (3.7%), *Geotrichum* (2.0%), *Wickerhamomyces* (2.0%) and *Penicillium* (0.7%). In DS, the reads were predominately assigned to *Saturnispora* (69.3%), followed by *Wickerhamomyces* (27.6%) and *Geotrichum* (3.1%), while in F, the reads were assigned to genera belonging to *Aspergillus* (45.4%), *Hyphopichia* (30.2%), *Cryptococcus* (10.2%), *Penicillium* (9.3%) and *Cladosporium* (4.9%).

## 4. Discussion

The use of selected starter cultures for dough fermentation is useful to obtain products with determined characteristics and to standardize the production processes [12,13,54]. Starters are known to have a strong impact on the microbial populations of doughs, consequently affecting product features. Therefore, the present work used the metagenetic method and the culture-dependent approach to analyze the biodiversity of doughs started with sourdough (type II) or baker’s yeast. It is well known that during traditional sourdough (type I) fermentation, the bacterial evolution is characterized by a decrease in Gram-negative and an increase in Gram-positive biota [55]. Ercolini et al. [29] observed flours with great diversity, mainly contaminated by genera (*Acinetobacter*, *Pantoea*, *Pseudomonas*, *Comamonas*, *Enterobacter*, *Erwinia* and *Sphingomonas*) belonging to the phylum *Proteobacteria*, although this population was almost completely inhibited after 1 day of sourdough propagation. On the contrary, although the phylum *Firmicutes* (mainly represented by LAB) was present at low relative abundances in the flours, it already became dominant after the first fermentation [29,55]. These microbial dynamics from flour to mature sourdough are well known and are in accordance with the microbial ecology of other fermented foods [16]. Flours analyzed in the present study were also found to be strongly contaminated by *Proteobacteria* (ca. 82%), and *Pseudomonas* dominated (49%) this microbiota. Contaminating bacteria originate from grain milling or are epiphytic and endophytic populations of wheat. In this study, sourdough type II (25%) or baker’s yeast (2%) was used as a single starter for dough leavening. The metagenetic analysis highlighted that, after fermentation, the *Proteobacteria* in DS were about 43.5% lower than in F and DB. At the same time, the flour population was replaced mainly by the starter strain. In fact, the metagenetic analysis showed that the *Leuconostoc* genus constituted 78.6% of LAB in DS. Moreover, as supported by the identification of cultivable LAB, *L. citreum* C2.27 was the only LAB strain isolated from DS.

On the contrary, *Proteobacteria* was the dominant phylum in F and DB samples. This phylum represents metabolically active populations, and the abundance of species belonging to the phylum *Proteobacteria* was mostly negatively correlated with dough and sourdough qualities [29,56]. It can be hypothesized that in dough with baker’s yeast, the fermentation conditions and the autochthonous microbiota of wheat flour, characterized, in particular, by the low LAB density, did not cause the reduction in *Proteobacteria,* which constituted, as in F, 82% of the bacterial population. In fact, the activity of the acid-producing bacterial starter during fermentation caused a rapid increase in the acidity of DS. Such an increase, together with other ecological parameters [16], may influence the microbial succession favoring LAB. On the other hand, LAB showed an increase within *Firmicutes* also in DB; in particular, *Lactococcus*, *Lactobacillus*, *Pediococcus* and *Leuconostoc* increased compared with F, but this was not sufficient to decrease the pH.

As described in other studies, *Staphylococcus*, *Streptococcus* and *Paenibacillus* had low ability to survive and colonize the doughs, especially DS. In fact, these genera are not generally found in doughs [17,31]. Regarding the presence of the genus *Leuconostoc* in F and DB detected by the metagenetic approach, it should be noted that this is probably due to the species *L. mesenteroides* isolated from DB together with *Fb*. *rossiae*, *Lp. paraplantarum* and *Lc. paracasei.* Metagenetic analysis also showed the presence of *Lactococcus* in the doughs, although it was not isolated from DS, probably due to the high starter load, or from DB, due to competition with other genera. In this regard, it should be considered that differences can be noted between metagenetic analysis and plating, especially for those LAB species that are generally difficult to cultivate [35].

Therefore, the flour plays a key role in establishing microbial consortia, but only species and/or strains adapted to the sourdough environment in relation to the nutrient availability and physic-chemical parameters of the process will grow and dominate in this ecosystem [17]. In type II sourdough, fermentation occurs after the inoculation of a starter culture. This starter culture can dominate and inhibit the growth of autochthonous dough microbiota because it is added in a high density [13]. However, the starter strain should be well adapted to the cereal environment in order to compete with the endogenous microbiota and be suitable for the process [57]. *L. citreum* C2.27, which was selected for its good leavening capacity, was isolated from durum wheat semolina and was suitable for the production process of yeast-free bread in the bakery [24]. In fact, it was the only strain isolated from liquid sourdough and DS, and the present study also confirmed its dominance by culture-independent analysis. This result is fundamental to guarantee the technological characteristics of the product made without using baker’s yeast, resulting, at the same time, in positive chemical-physical and nutritional characteristics of the dough and final product, as determined in a previous work [27].

The results show that yeasts were present in very low quantities in DS and F. They are normally associated with flours up to 3.3 log cfu/g [57], and the genera were those typically found in flours and doughs [17,24,58]. *Ascomycota* was the only phylum present in doughs, but not in the flours evaluated. The mycobiota changed from flour to doughs; in fact, the genera *Hyphopichia* and *Cryptococcus* were found only in F, and the latter was also the only genus isolated directly from F. Considering the mycobiota of flours, molds were dominant in F, but, overall, in very low quantities. Therefore, the metagenetic approach made it possible to obtain more information on the mycobiota composition even if present in small numbers.

The absence of *Saccharomyces* in F and DS was interesting. *S. cerevisiae* is the species of yeast most frequently isolated in sourdoughs from central and southern Italy [52]. However, several studies have shown different compositions of yeast species between artisanal bakery and spontaneous laboratory sourdoughs and, in particular, have hypothesized the presence of *S. cerevisiae* in bakery sourdoughs due to contamination of the bakery environment with commercial baker’s yeast [58,59]. Conversely, as observed in the present study, yeasts detected in sourdough and doughs made in the laboratory could only come from flours [24,60].

Finally, the results for DS, which was dominated by *L. citreum* C2.27, also show a reduction in the fungal diversity, probably related to the antifungal properties of the starter strain [36], while DB, with a *S. cerevisiae*-dominated mycobiota, presented a greater bacterial diversity.

## 5. Conclusions

This study showed how the use of a microbial starter deeply affects the composition of the dough microbiota, which is directly responsible for the quality of the product. To our knowledge, this work is the first that analyzes, also using the metagenetic method, the bacterial and fungal microbiota of doughs leavened with baker’s yeast or type II sourdough (started with a selected bacterial strain), also making it possible to verify the absence of *S. cerevisiae* in the latter used to obtain an innovative “yeast-free” product.

Metagenetic analyses indicated that the V5–V6 hypervariable regions of the 16S rRNA gene and the V9 hypervariable region of the 18S rRNA gene were suitable for studying the microbiota of doughs, providing a comprehensive overview of the microbial community. The culture-independent approach allowed gaining deeper and wider knowledge of the starter impact on the microbial populations of the doughs, even if the association with the culture-dependent analysis is still very useful to obtain more precise information at the species level on certain microbial categories. This study highlighted that, differently from doughs produced with the *L. citreum* starter, the dough made with baker’s yeast contained a microbiota with a high abundance of *Proteobacteria* (82% of the bacterial population), which were negatively correlated with the biochemical properties of the doughs [27,56]. Furthermore, the analyses showed the ability of the *L. citreum* C2.27 starter to dominate the microbiota, also inhibiting the growth of *S. cerevisiae*. This result is particularly important because *L. citreum* C2.27 has been adopted for its leavening abilities in a biotechnological protocol for the production of “yeast-free” bakery products.

## Figures and Tables

**Table 1:** Acidification (ΔpH, pH units), total titratable acidity (TTA, ml di NaOH 0.1 N/10 g) and microbiological characteristics of durum wheat semolina and soft wheat flour type “00” in a 1:1 ratio (F), liquid sourdough (S), doughs started with the liquid sourdough (DS) and dough made with baker’s yeast (DB).

Sample	pH	ΔpH	TTA	LAB (log cfu/g)	Other LAB Species Isolates	Spore-Forming Bacteria (log cfu/g)	Yeasts (log cfu/g)	Molds (log cfu/g)	Yeast Species
F	-	-	-	0.46 ± 0.28	*Ll. coryniformis*	0.8 ± 0.72	0.7 ± 1.15	2.59 ± 0.12	*Cr. victoriae*
S	3.53 ± 0.07	2.42 ± 0.14	4.15 ± 0.21	9.47 ± 0.13	nd	nd	nd	1.57 ± 0.04	nd
DS	4.23 ± 0.04 ^b^	1.35 ± 0.09 ^a^	8.17 ± 0.55 ^a^	9.09 ± 0.01 ^a^	nd	0.54 ± 0.08	nd	2.70 ± 0.36	nd
DB	5.38 ± 0.02 ^a^	0.25 ± 0.01 ^b^	3.55 ± 0.21 ^b^	4.48 ± 0.08 ^b^	*Fl. rossiae* *L. mesenteroides* *Lp. paraplantarum Lc. paracasei*	nd	8.25 ± 0.01	nd	*S. cerevisiae*

Data represent means of three independent experiments ± standard error. ^a–b^ Values referring to *focaccia* dough in the same column with different letters differ significantly (*p* < 0.05). *Ll*., *Loigolactobacillus*; *Fl*., *Furfurilactobacillus*; *L*., *Leuconostoc;* *Lp*., *Lactiplantibacillus*; *Lc.*, *Lacticaseibacillus*; *Cr.*, *Cryptococcus; S*., *Saccharomyces*. nd: not detected (<LOD, limit of detection).

## Data Availability

The sequence data are available at NCBI SRA under BioProject ID: PRJNA668040.
